# American Medical Association

**Published:** 1884-06

**Authors:** John S. Marshall


### AMERICAN MEDICAL ASSOCIATION.

### SECTION OF ORAL AND DENTAL SURGERY.

Reported for the Independent Practitioner

BY JOHN S. MARSHALL, M. D.

The thirty-fifth annual meeting of the American Medical Association was held in Washington, D. C., May 6th to 9th inclusive, and was largely attended, over 1,300 names being registered. The section on Oral and Dental Surgery was well represented. Its meetings were held in the afternoon from 2.30 to 5 o'clock; the mornings being devoted to the sessions of the general body. The papers read before the section were as follows:

"On Assuring Healthy Dentine over Endangered Pulps," by Jacob L. Williams, M. D., Boston, Massachusetts.

"Dental Caries and its relations to the Germ Theory of Disease," by G. V. Black, M. D., Jacksonville, Illinois.

"Sponge Grafting," by Edward C. Briggs, M. D., Boston, Massachusetts.

"The Removal of Stains from the Teeth Caused by the Administration of Medicinal Agents, and the Bleaching of Pulpless Teeth," by A. W. Harlan, M. D., Chicago, Illinois.

"Overdraught of Nervous or Vital Power as Affecting General and Special Health," by Jacob L. Williams, M. D., Boston, Massachusetts.

"Periodic Hemorrhage from the Gums, Associated with Recession of the Gums and Alveolar Processes, the result of Amenorrhœa," by W. W. Allport, M. D., Chicago, Illinois.

The first session was called to order by the chairman, Truman W. Brophy, M. D., of Chicago, Illinois, who after a few words of welcome called for the reading of the paper of Jacob L. Williams, M. D., of Boston, Massachusetts, "On Assuring Healthy Dentine over Endangered Pulps."

The author began by describing what he wished to be understood as meaning by *endangered* pulps, viz.: those cases in which the pulps through the action of caries have but little of the natural covering of dentine left over them, and which covering is very apt to be more or less tainted with the agents that have produced the pathological condition.

In such cases there will be found an acid condition, with fermentive action often in full sway, demanding at a first thought the most positive and effective neutralizing and corrective applications. Just beyond, however, lie some of the most highly sensitive and delicately organized tissues of the whole body; fibers that if rudely handled will set in motion vibrations that will be likely to continue, or be repeated in a very serious way; tissues which if once inflamed, none are more doubtful of survival. The preservation of the vitality of the pulps in these cases means additional years of usefulness of the tooth. These circumstances are often overlooked, or not taken into consideration in using violent or caustic applications. He then asked the question, "What should be done?" and stated that his ideas upon this point might best be given by mentioning his plan of treatment which was formed, tested, and adopted during the years 1847–1848, and which in general principles he still found most satisfactory up to the present time, though new materials had been added to the list of remedies for diseased conditions since that date.

The plan of treatment is as follows: After removing the loose debris he applied a mild antacid, such as aqua calcis, or a solution of bicarbonate of soda, after which was applied a solution of chloride of calcium to neutralize the fermentive elements, and disinfected with very dilute wood creosote. The cavity was then dried and sealed up for from two to three weeks, after which it was again opened and subjected to the same treatment, with the addition of a mild astringent, such as tannic acid in weak solution, and again sealed, generally for a longer period. This dressing to be repeated at proper intervals and continued until permanent health of the dentine was assured; sometimes extending over a period of two years or more.

The object of this plan of treatment is to avoid the undue irritation of the pulp so likely to follow the application of the stronger remedies, and which often results in serious mischief. With the mild treatment the effect is more or less transient and requires frequent repetition, but this will be required less often as the dentine gains in soundness and health. By this method we help nature in the line of her preference in protecting rather than injuring a vital point, and keep the disease in check. He gave as his observation and practical experience that the proportion of successful cases from the "mild repetition" plan was far beyond that of the single capping practice. Every attempt, however, will not be crowned with success; constitutional as well as local causes, both sometimes of an obscure nature, may thwart our plans; the neglect of the patient, or his piratical appropriation by some other practitioner who does not appreciate the object of the treatment, may cause a failure.

The paper closed with a detailed description of a representative case, and the treatment adopted was substantially that given above.

DISCUSSION.

*Dr. W. W. Allport,* Chicago, Ill.—Was certainly very much interested in the paper and the line of treatment marked out by the author. It has been the usual practice to apply antiseptics in these cases—creosote, carbolic acid, eucalyptus, etc., in various strengths, and then to immediately cap with some non-conducting material like gutta-percha, oxy-chloride or oxy-phosphate of zinc.

The first important point to be gained is to neutralize or destroy the agents of decay, and then to protect the pulp from irritation of every variety. This can best be done by using a non-conducting material directly over the pulp, and then to fill the balance of the cavity with a substance sufficiently hard and firm to prevent irritation from mechanical pressure. But we do not see why the treatment need be continued over so long a period as that practiced by Dr. Williams.

*Dr. J. L. Williams*—The object in continuing the treatment over so many months is to be sure that the active causes of decay are destroyed or rendered inert, and to guard against pulp irritation by frequent dressing with mild remedies, rather than to run the risk incident to the application of such intense medicaments as are commonly used.

*Dr. Allport*—I have heard something recently about a new remedy for the treatment of this class of diseases, made from the oil of cloves, and known as Eugenol. It has been very highly recommended as having a decidedly fatal influence over germ life. Have had no experience with it myself, but would like to hear a report upon the subject from those gentlemen who have experimented with it.

*Dr. A. W. Harlan*, Chicago, Ill.—Dr. Williams spoke of his treatment as antagonizing the acid or acid ferment. Such treatment could be only of temporary benefit; the remedies must be of sufficient strength to destroy the agents of fermentation. Most practitioners are in the habit of swabbing the cavity of decay with creosote, carbolic acid, and other like remedies, and after temporarily sealing it up for a few days, capping with gutta-percha solution, oxy-chloride of zinc, metal caps, with various forms of dressing placed upon the under surface, and permanently filling the cavity of decay.

But in such cases we have no means of knowing whether there has really been any deposit of secondary dentine, and I believe in a vast majority of them no secondary deposit occurs.

Dr. Allport moved the further discussion of the paper be deferred until to-morrow, as he had been informed that Dr. G. V. Black, of Jacksonville, Ill., would present a volunteer paper upon a subject closely allied to the one under discussion, and it would be very desirable to consider them both together. Motion was carried. Dr. John S. Marshall, Chicago, Ill., made a motion inviting Dr. Black to read his paper to-morrow afternoon, and that it be the first order of business. Motion carried. The next essay on the programme was by Dr. Reuben A. Vance, of Cleveland, Ohio, "On the Removal of Tumors from the Upper Jaw," but Dr. Vance not being present the subject was passed. On motion of Dr. Allport a committee on publication was appointed, to whom all papers must be referred. Committee, Drs. Allport, Marshall, Harlan.

Section adjourned to meet on Wednesday at 2.30 P. M.

(TO BE CONTINUED.)

